# 

**Published:** 1883-02

**Authors:** 


					Alabama State Dental Association.
The Fourth Annual Meeting of the Alabama State Den-
tal Association, will be held in Montgomery, Alabama, on
the second Tuesday in April, 1883
All are cordially invited, and an interesting programme
may be anticipated.
E. WAGNER, D. D. S., Secretary.
P. O. Box 138. Montgomery, Alabama.
The Annual Commencement of the Dental Department of the
University of Maryland, will be held at the Academy of
Mu&ic, Baltimore Wednesday, March 15th, 1883. The Dental
ProfeHsion i& invited to attend.
To Readers and Correspondents.
Communications intended for publication, and exchange journals and p
pers, should be addressed to the Editor of the American Journal of De]
tal Science, 259 N. Eutaw St., Baltimore, Md. All letters relating to
business of the journal, or containing cash remittances,, to be directed
Messrs. Snowden & Cowman. Articles for publication should be received
the editor at least three weeks previous to the first of the month on which t
number in which it is designed for them to appear, is issued.
^gTThe American Journal of Dental Science will contain fifty pagej
of reading matter in each number, making a volume of about
Six Hundred pages,
EXCLUSIVE OF ADVERTISE MP] NTS.
T 11 K
AMERICAN JOURNAL
OF
DENTAL SCIENCE.
The Journal is issued on the first of each month and contains not less
than fifty pages of reading matter in each number, exclusive of adver-
tisements, making a volume of six hundred pages per annum.
In each number will be found Original Communications, Correb-
pondence, Selected Articles, Monthly Summary, Bibliographical No-
tices and Editorial Matter, and every effort will be exerted to render
the Journal worthy of the patronage of the Dental profession.
A generous co-operation is respectfully solicited from the members
of the profession ; and as the Journal has now a printing office of its
own, which materially lessens the cost of its publication, it will be
issued at the reduced subscription price of
$2.50 Per Aiiuum in Advance.
Communications are respectfully solicited on all subjects pertain-
ing to the practice of dentistry, as well as reports of cases occurring
in dental practice.
HATES OF ADVERTISING.
1 MO.
1 Page. $15 00
i " | 10 00
1 ? | 7 00
I " 5 0.0
2 MO.
$22 50
15 00
11 00
8 00
3 MO.
$30 00
20 00
15 00
11 00
6 MO. I 12 MO.
$50 00
30 00
20 00
15 00
All original communications designed for publication, works for
r3?iew, new instruments for notice, exchanges, &c., should be directed
to the editor, 259 N. Eutaw St., Baltimore, Md.
All advertisements and subscriptions should be addressed to
SNOWDEN & COWMAN,
Publishers of the Am. Journal of Dental Science.
86 W Fayette St. Bet. Charles & Liberty Sts.,
BALTIMORE, MD

				

